# Combining *in vitro* and Field Studies to Predict Drought Tolerance in *Vicia sativa* L. Genotypes

**DOI:** 10.3390/plants14213376

**Published:** 2025-11-04

**Authors:** Juan M. González, Yolanda Loarce, Noa Sánchez-Gordo, Lucía De la Rosa, Elena Ramírez-Parra

**Affiliations:** 1 Departamento de Biomedicina y Biotecnología, Universidad de Alcalá, Campus Universitario, 28805 Alcalá de Henares, Spain; yolanda.loarce@uah.es (Y.L.); noa.sanchez@edu.uah.es (N.S.-G.); 2 Centro de Recursos Fitogenéticos, Instituto Nacional de Investigación y Tecnología Agraria y Alimentaria, Consejo Superior de Investigaciones Científicas (CRF-INIA/CSIC), 28805 Alcalá de Henares, Spain; lucia.delarosa@inia.csic.es; 3 Centro de Biotecnología y Genómica de Plantas, Instituto Nacional de Investigación y Tecnología Agraria y Alimentaria, Consejo Superior de Investigaciones Científicas (CBGP, UPM-INIA/CSIC), Universidad Politécnica de Madrid, Campus de Montegancedo, 28223 Pozuelo de Alarcón, Spain; ramirez.elena@inia.csic.es

**Keywords:** root, shoot, proline, PEG, drought stress, common vetch

## Abstract

Vetch (*Vicia sativa* L.), an important forage legume, faces increasing drought stress due to climate change. This study evaluated drought responses in 26 genotypes using both in vitro and field trials. *In vitro* experiments analysed seedlings grown on culture media either with 20% polyethylene glycol (PEG) to simulate drought (C20) or without PEG as a control (C0), measuring root and shoot dry weights as well as proline content. Field trials under rainfed and drought conditions assessed 100 seed weight and seed weight per plant. All traits studied exhibited high variability, with elevated coefficients of variation and broad-sense heritability. Seedling roots grown in C20 had higher dry weight than those in C0, while shoots showed the opposite trend. In C20 medium, proline content increased significantly—by 118.1% in roots and 131.1% in shoots. However, proline concentration did not correlate with field yield traits, limiting its utility as a drought tolerance marker. Principal component analysis grouped genotypes based on biomass production and drought response. Importantly, *in vitro* root and shoot dry weights were positively correlated with field yield traits, indicating their value as early predictors of agronomic performance and offering a useful tool for selection in vetch breeding programmes.

## 1. Introduction

The United Nations estimates that the world’s population will reach between 9.1 and 9.7 billion by 2050 [1,2]. This growth will require a significant increase in protein production, which is expected to grow by 50% to meet the food needs of both humans and livestock. Conventional sources of animal protein exert considerable pressure on the environment, meaning a transition to more sustainable alternatives is required. Legumes represent a promising option due to their high protein content, along with their symbiotic association with nitrogen-fixing bacteria (e.g., *Rhizobium*) that improve soil fertility and reduce dependence on synthetic fertilisers; thus, these crops play a key role in promoting ecological balance in agriculture [3,4]. Furthermore, their adaptability to limited water availability supports cultivation in dry environments, expanding agricultural potential in arid regions [5,6].

Drought is one of the most limiting environmental factors, leading to reduced agricultural productivity [7,8,9,10]. Water stress leads to an increase in reactive oxygen species (ROS) [11] and the accumulation of compatible solutes such as proline, and therefore, their concentration could be used as an indicator of drought tolerance [12,13,14].

It should be noted that estimating drought tolerance in the field can be resource-intensive; therefore, the development of efficient phenotyping systems in controlled environments for early assessment can be a useful tool for the evaluation of large collections. Thus, laboratory techniques, such as *in vitro* methods using polyethylene glycol 6000 (PEG 6000), as an osmotic agent used to simulate drought stress in plants, have proved effective in the study of different species including lentil, chickpea, soybean, or tomato, and they can be utilised to identify genotypes with different levels of drought tolerance prior to field trials [14,15,16,17,18]. In addition, assessing how plants respond to water stress during the early stages of growth could help to understand their resilience. This phase is critical to the success of plant establishment, varies among genotypes, and may be indicative of final crop yield, as some authors have shown [19,20,21,22]. Seedling growth depends on the development of roots and shoots. Root system size influences the efficiency of water and nutrient uptake, while shoot growth reflects the potential accumulation of aerial biomass and can be a predictor of future plant performance [23].

Within the family *Leguminosae*, the genus *Vicia* comprises between 210 and 240 species [24], with common vetch (*Vicia sativa* L.) being one of the most economically important ones [25]. This species is highly valued for its high protein and mineral content, as well as for its energy value, which makes it a valuable resource for both feed and fodder [26,27]. Its capacity to fix atmospheric nitrogen supports soil enrichment and minimises dependence on synthetic fertilisers, reinforcing its role in sustainable agricultural systems [28]. The broad adaptability of *V. sativa* is reflected in its extensive cultivation across diverse climatic zones, ranging from tropical and subtropical to temperate regions [29,30]. Despite the agronomic interest of vetch, few commercial varieties are adapted to the current context of climate change. One of the objectives of genetic improvement of vetch is to develop drought-tolerant varieties, since this species is mainly grown in arid/semiarid areas under rainfed conditions. Regarding this stress in *V. sativa*, recently, some molecular and biochemical mechanisms involved in drought tolerance are beginning to be elucidated [27]. Transcriptomic studies have revealed genes and pathways related to stress response, including those regulating stomatal conductance, reactive oxygen species dynamics, and osmoprotectant synthesis [25,31]. However, the complexity of genetic variation and the inconsistent expression of these traits under field conditions have so far limited the precise selection of reliably drought-tolerant genotypes. Thus, while molecular markers and gene expression profiles offer promising tools, practical breeding for drought resilience in common vetch is still a significant challenge and the identification of clearly tolerant *V. sativa* genotypes remains elusive.

Usually, breeding programmes start with the study of the genetic variability of the traits associated with the target improvement objective in large collections of genotypes. This allows for the selection of the most suitable genotypes for crossbreeding, from which the lines best fitted to the desired objectives are selected. Research and breeding efforts aimed at increasing crop resistance are facilitated by genebank collections, which play a crucial role in this regard [32,33,34]. The evaluation of many genotypes would be facilitated using biomarkers that are easily quantifiable by simple and rapid assays and that correlate with relative tolerance to water deficit stress. Proline, a common plant osmolyte, could be an appropriate candidate because significant increases in proline content in response to water deficit or other stressful conditions have been detected in many species [13,35,36,37].

The objective of the present study is to assess the genetic variability associated with drought stress tolerance in *V. sativa*, using different experimental approaches. A collection of 26 genotypes, including commercial varieties, landraces, and wild accessions, will be analysed. Root and shoot dry weight from *in vitro* grown seedlings will be measured under standard culture conditions and in a medium supplemented with 20% polyethylene glycol (PEG-6000) to induce water stress. Additionally, proline concentration will be quantified in fresh root and shoot tissues of plantlets of the same genotypes and culture conditions. The results will be compared with key agronomic traits of vetch (100 seed weight and seed weight per plant) in plants grown under two experimental conditions: rainfed and in a rain shelter to induce higher drought stress. Identifying potential correlations between the traits analysed across different experiments will provide insights into whether seed yield can be predicted at early developmental stages, offering a time- and space-efficient approach for vetch breeding programmes.

## 2. Results

A total of 22 variables were studied in *in vitro* and field experiments. The description of the variables and abbreviations used throughout the text are shown in Table 1. The mean values and standard deviations for each of the 22 traits analysed for each of the 26 vetch genotypes studied are shown in Table S1 of the Supplementary Materials.

### 2.1. Genotypic Variation in Root and Shoot Biomass Under Osmotic Stress

**Figure 1:**
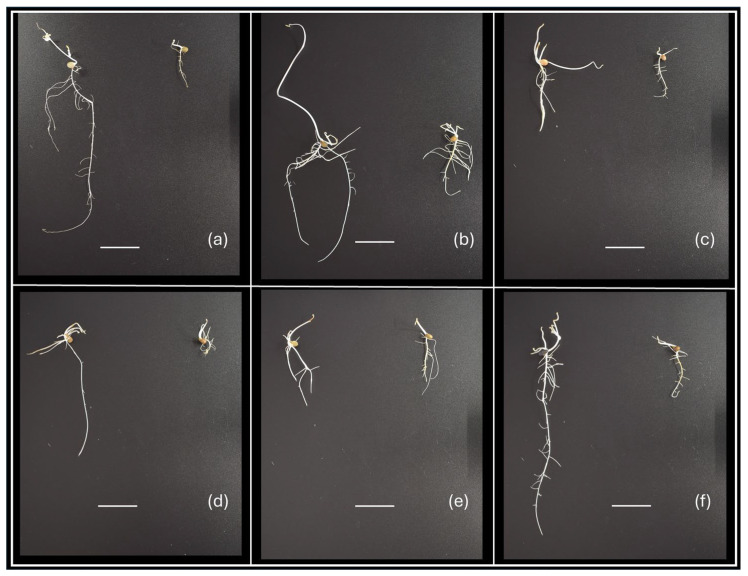
Examples of phenotypic variability of vetch seedlings after 15 days of *in vitro* culture, showing differences in shoot and root development according to genotype and C0 or C20 culture medium used. Genotypes: (**a**) V9; (**b**) V8; (**c**) V21; (**d**) V14; (**e**) V17; and (**f**) V5. In each image, the seedling on the left corresponds to growth in C0 medium and the one on the right to growth in C20 medium. The horizontal white line is 2 cm long.

To evaluate the effect of drought on vetch accessions through *in vitro* experiments, two approaches were used. One was the analysis of the dry weight of roots and shoots of seedlings grown in C0 or C20 medium, and the other was the analysis of the proline concentration in roots and shoots of seedlings grown in the same media. Phenotypic differences in growth were observed between genotypes and culture media used (Figure 1).

**Figure 2:**
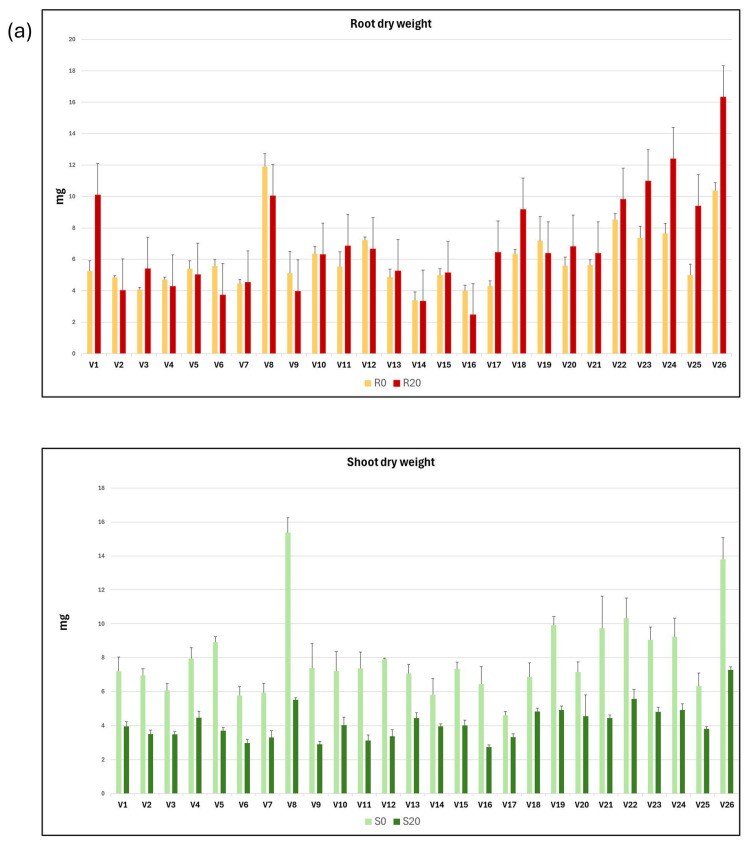

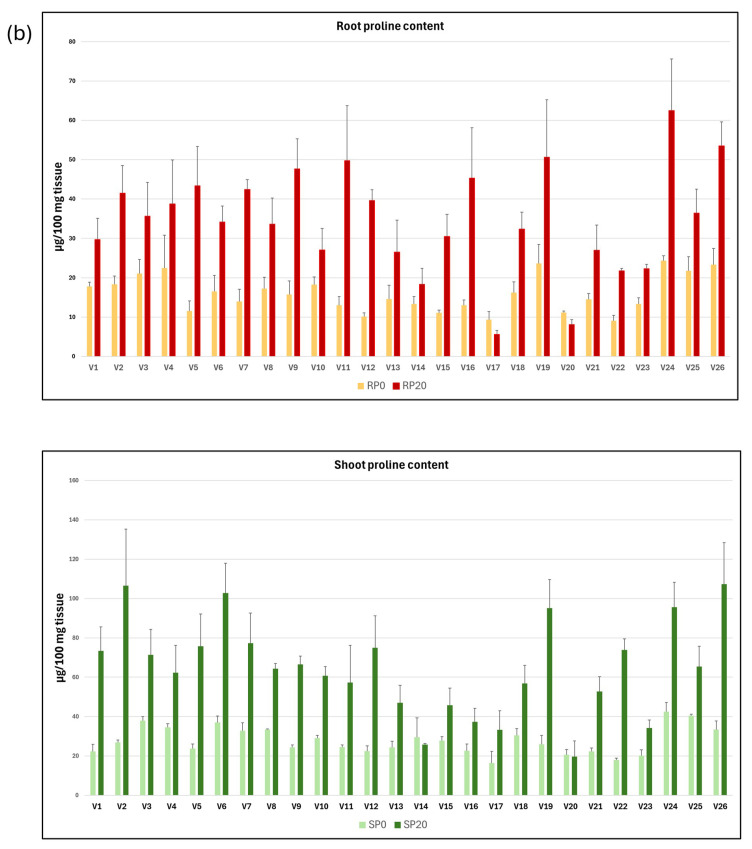
Graphical representation of the mean and standard deviation of root and shoot dry weight (**a**) and proline content (**b**), both in C0 and C20 media, for each of the 26 vetch genotypes studied.

The mean values and standard deviation of each of the variables related to dry weight and proline concentration in roots and shoots from seedlings grown in C0 and C20 media are shown as graphs in Figure 2a,b.

A statistical summary of the intergenotypic variation and broad heritability of traits related to root and shoot dry weight and proline concentration is presented in Table 2. On average, root dry weight in C20 increased by 16.4% compared with C0, while the dry weight of the shoots developed in C20 medium decreased by 47.9% compared with C0 medium. The R20/S20 ratio was 122.7% higher than the R0/S0 ratio, and the R0/R20 and S0/S20 ratios were 0.86 and 1.92, respectively. These results indicate that while the dry weight of roots increases in C20 medium compared with C0 medium, the dry weight of shoots decreases.

The proline content of roots and shoots developed in C20 medium increased significantly compared with those developed in C0 medium, with increases of 118.1% and 131.1%, respectively. The proline mean concentration of the shoots relative to that of the roots developed in the C0 (RP0/SP0) and C20 (RP20/SP20) media were 0.58 and 0.56, respectively, indicating that almost twice as much proline is accumulated in the shoots as in the roots.

It is noteworthy that a high degree of genetic variability was observed among the genotypes, with a wide range between the maximum and minimum values for each trait and a coefficient of variation (CV) ranging from 46.31 to 14.9 for R20 and R20/S20, respectively. For the traits analysed, the broad sense heritability (h^2^%) was high, ranging from 30.7% for RP0/RP20 to 90.3% for R0, indicating that the variation found between genotypes has a wide genetic basis.

### 2.2. Seed Yield Decreased Under Drought, with Strong Genotypic Variation

**Figure 3:**
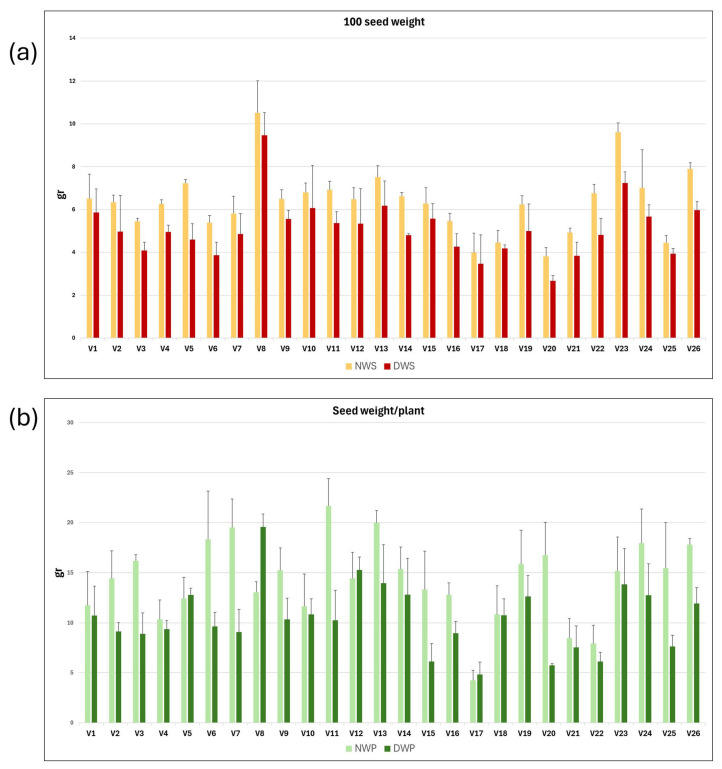
Graphical representation of the mean and standard deviation of 100 seed weight (**a**) and seed weight per plant (**b**) in rainfed (N) and drought (D) conditions for each of the 26 vetch genotypes studied.

Vetch plants grown under rainfed (N) and drought (D) conditions, respectively, were harvested at maturity. The weight of 100 seeds and the average seed weight per plant were measured for each of the three replicates of the 26 vetch genotypes. Mean values and standard deviations for NWS, DWS, NWP, and DWP traits; and for the derived variables NWS/DWS and NWP/DWP, for each vetch genotype, are presented in Table 2. The mean values and standard deviation of NWS, DWS, NWP, and DWP variables are shown as a graph in Figure 3a,b. It can be observed that water stress reduced seed production parameters, resulting in a decrease in both 100 seed weight and seed weight per plant across the different genotypes.

The intergenotypic variability has been studied, and the mean, maximum and minimum values, standard deviation, coefficient of variation, and broad heritability are shown in Table 2. As expected, higher mean, maximum, and minimum values were observed for plants grown under natural conditions, compared with plants grown in the rain shelter. Thus, the 100 seed weight and seed weight per plant of plants grown under natural field conditions were 19.3% and 36.6% higher than those grown in drought conditions.

### 2.3. Significant Genotype and Environmental Effects on the Analysed Traits

Analyses of variance were performed for each of the 22 traits studied, taking genotype as an independent factor, and the results were statistically significant in all cases (*p* < 0.01). Table S2 of the Supplementary Materials presents the results of Tukey’s HSD multiple range tests for each of the traits, showing which genotypes exhibited statistically significant differences. For some traits, such as R0/S0, it was observed that the 26 genotypes can be assigned to 4 homogeneous groups, while for others, such as R20/S20, the genotypes could be grouped into 11 different homogeneous groups, which would indicate a great diversity in the behaviour of the genotypes for this trait.

**Figure 4:**
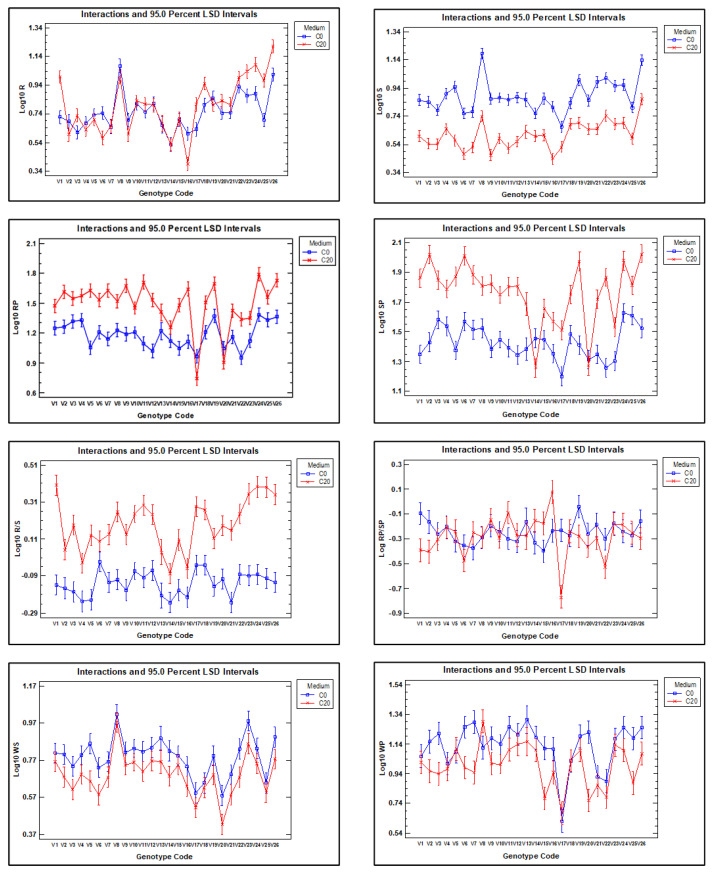
Interaction plots for each of the eight dependent traits analysed in the multifactorial ANOVA, illustrating the effects of the growth medium (C0 and C20) for *in vitro* experiments or natural (N) and drought (D) conditions for field experiments, on the 26 vetch genotypes. (R) Root dry weight (S), shoot dry weight (R/S), root-to-shoot dry weight ratio (RP), proline concentration in roots and shoots (SP), root-to-shoot proline concentration (RP/SP). (WS) The weight of 100 seeds; (WP) the average seed weight per plant. The 95% confidence interval of LSD is indicated for each data.

To assess the effect of genotype and growing medium on traits related to dry weight and proline concentration, multifactorial ANOVAs were performed using the following traits: root dry weight (R), shoot dry weight (S), root-to-shoot dry weight ratio (R/S), proline concentration in roots (RP), proline concentration in shoots (SP), and root-to-shoot proline concentration (RP/SP). The same type of analysis was performed for the traits 100 seed weight (WS) and seed weight per plant (WP) as a function of genotype and whether the plants were grown under normal field or drought conditions. The model included the main effects of both factors and their interaction. The results revealed statistically significant differences (*p* < 0.01) for all traits across all evaluated effects, except for the medium factor in RP/SP and the GxM interaction in WS (Table 3), and Figure 4 shows the interaction plot for the seven traits.

These results confirm that root dry weight was generally higher in seedlings grown in C20 medium than in C0, although the difference was statistically significant only for genotypes V1, V3, V6, V17, V18, V23, V24, V25, and V26, while for genotype V16, root dry weight was significantly higher in C0 than in C20. In contrast, shoot dry weight was consistently higher for all genotype growths in C0 than in C20, indicating greater sensitivity of shoot biomass production to water stress in C20. The root-to-shoot dry weight ratio was higher in all genotypes when grown in C20 compared with C0, which confirms that water stress affects shoots more than roots. However, it is important to note that the response of vetch genotypes is highly variable. For example, some genotypes show a marked difference in the R/S ratio between the two growth media (e.g., genotypes V1, V11, V23, V24, V25, and V26), while for genotypes V6, V14, and V16, the difference between the two media was much smaller.

It is observed that the proline concentration is higher in C20 medium than in C0 medium, except in the roots of genotypes V17 and V20 and in the shoots of genotypes V14 and V20.

Hundred-seed weight (WS) and seed weight per plant (WP) were higher in all genotypes when plants were grown under natural conditions than when they were grown in the rain shelter under drought conditions, although the differences were statistically significant only for some of them, such as V3 and V26. Furthermore, in the case of the V8 genotype, the seed weight per plant was higher in plants grown in the rain shelter than in those grown under natural conditions.

### 2.4. Correlations Between in vitro and Field Traits

**Figure 5:**
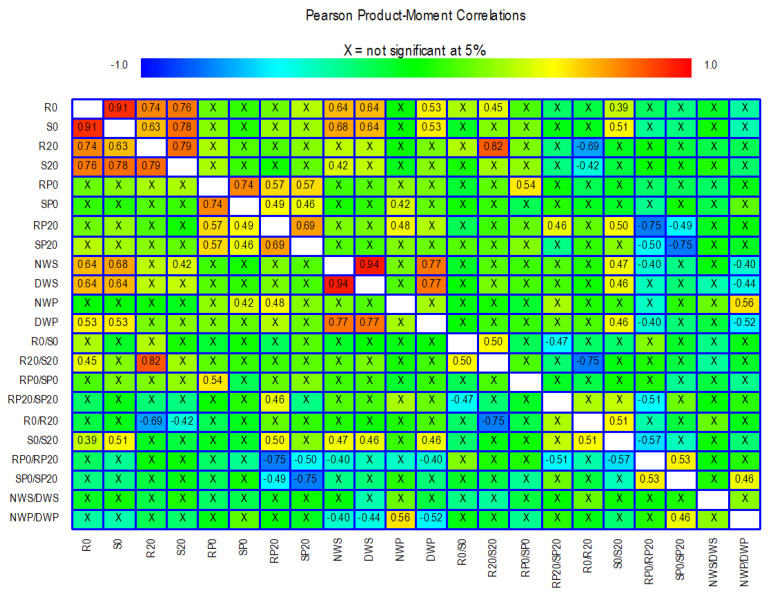
Results of Pearson’s correlation tests among the 22 traits analysed. Correlations that are not significant are marked with an X, and those that are significant are marked with a numerical value. Colours are used to highlight the magnitude of the correlations with a range from −1 to +1. The meaning of the abbreviations and the description of each trait is shown in Table 1.

Pearson’s correlation coefficients were calculated to assess the relationships between the 22 traits analysed in the *in vitro* and field experiments (Figure 5).

Some of the traits related to root and shoot dry weight showed high positive correlations between them, e.g., R0, S0, R20, and S20. In contrast, the traits derived from dry weight showed less significant correlations with each other and with the other dry weight traits. For example, the R0/S0 ratio was not significantly correlated with any other trait except the R20/S20 ratio (r = 0.50). For the 100 seed weight and seed weight per plant traits, there are correlations with some of the shoot and root dry weight traits. Particularly noteworthy are the correlations between R0 and S0 with the traits NWS, DWS, and DWP. The traits related to proline concentration correlate with each other while they hardly correlate with the remaining traits. For example, RP20 has a correlation of 0.5 with the trait S0/S20. Correlations between SP0 and RP20 with NWP are also observed, although they do not reach 0.5.

### 2.5. PCA Grouped Vetch Genotypes by Contrasting Biomass Production and Drought Tolerance

Considering that the traits related to proline content in roots and shoots scarcely show correlations with the rest of the traits and that, in vetch, both the aerial part is of interest for its biomass production, mainly seeds, and the roots for nitrification and soil fixation, it was decided to perform a PCA with the 14 traits related to biomass production both *in vitro* and in the field to determine their contribution to the separation of the 26 vetch genotypes studied. Table 4 shows the weight of each trait in the first two principal components, which account for 38.2% and 21.1%, respectively, of the variance total.

**Figure 6:**
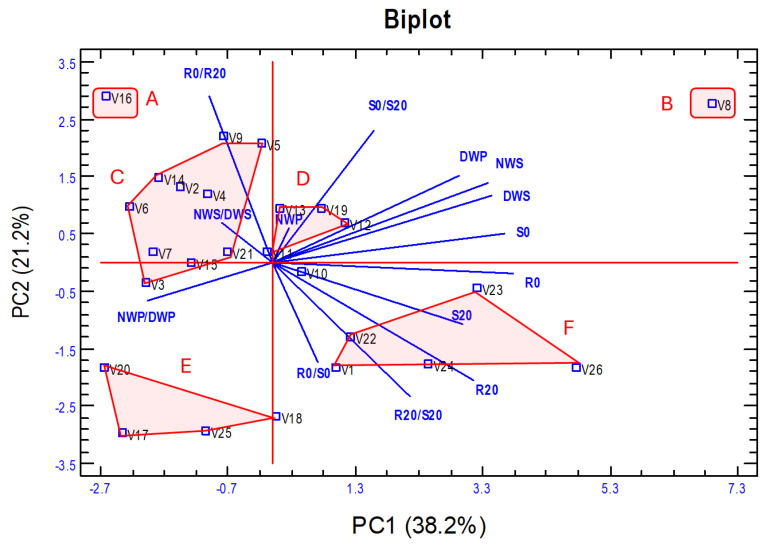
Biplot of the first two principal components of the PCA analysis from the 14 traits analysed in the 26 vetch genotypes. Six groups (A–F) of genotypes have been established based mainly on their biomass production (PC1) and tolerance to water stress (PC2). The meaning of the abbreviations and the description of each trait is shown in Table 1.

The variation in PC1 was primarily determined positively by the dry weight of roots and shoots in seedlings grown in C0 or C20 medium (R0, S0, R20, S20) and 100 seed weight (NWS, DWS) and plant seeds weight (DWP) and negatively by NWP/DWP. PC2 was mainly determined positively by the dry weight ratios of roots and shoots under control versus hydric stress (R0/R20, S0/S20), DWP, and NWS and negatively by R20, R0/S0, and R20/S20. Figure 6 presents a biplot of the first two principal components, showing the vectors of the traits and the vetch genotypes scattered on the two axes of the biplot.

Based on the PCA analysis, the vetch accessions were classified into six groups (Figure 6), and a statistical summary of the values of each of the 14 traits in each of the established groups is shown in Table 5. It should be noted that the CVs are low for all traits, which shows that the proposed groups are formed by rather homogeneous vetch genotypes. Group A includes only the V16 genotype and is characterised by very low biomass production and the lowest tolerance to water stress. Group B includes genotype V8, characterised by low tolerance to water stress, but has the highest biomass production. Group C includes 10 genotypes that have low–medium values for both biomass production and tolerance to water stress. Group D includes five genotypes with medium values for biomass production and tolerance to water stress. Group E includes four genotypes with low biomass production but has the highest level of hydric stress tolerance. Group F, with five genotypes, has medium–high values for biomass production and hydric stress tolerance. In these two last groups, the R0/S0 and R20/S20 ratios are relatively high, indicating a higher root development in relation to shoot development.

## 3. Discussion

There is currently an urgent need to develop new plant varieties capable of maintaining or even increasing agricultural productivity, which is being severely impacted by climate change. Water stress is one of the most limiting environmental factors for plant development, especially in the juvenile stage, as it drastically alters the essential physiological processes necessary for plant growth, reducing agricultural productivity [9,14,16,18,38]. Research on the effect of drought on plant growth is mainly based on the comparison of biomass production under water stress relative to an unstressed control [39,40]. In this context, estimation of drought tolerance in vetch and other crops is resource-intensive, so the development of efficient phenotyping systems at early stages and in controlled environments could be of great interest [41]. The use of polyethylene glycol (PEG) to simulate drought stress under *in vitro* conditions provides a controlled method of evaluating drought tolerance in seedlings and distinguishing tolerant genotypes [15,16,17,18,22,42]. Since seedling development and vigour can determine subsequent plant development and yield, it is important to study the effects of drought at this stage, as Alicandri et al. [43] showed in a study on bean; for this species, morpho-physiological traits measured in juveniles could predict drought tolerance in adults.

The availability of broad genetic variation is the most important prerequisite for improving drought tolerance in vetch. Furthermore, identifying vetch accessions at the extremes of the drought tolerance spectrum will enable further investigation into the underlying mechanisms of tolerance to this abiotic stress. In the present work, both *in vitro* experiments using a control medium (C0) and another including 20% PEG to induce water stress (C20), as well as field cultivation experiments under natural and drought conditions, showed significant variability in the 26 vetch genotypes analysed (Table S1, Table 2, Figure 2 and Figure 3).

### 3.1. In vitro Studies

We observed that, on average, seedling roots grown in C20 medium exhibited a greater dry weight compared with those grown in C0 medium, whereas the shoots displayed the opposite trend (Table 2). PEG treatments have been shown to trigger adaptive changes in root architecture, helping plants to access more water under simulated drought conditions by increasing root length and diameter and sometimes total root dry weight. Furthermore, this response may be genotype-specific, indicating that certain genotypes have greater adaptive plasticity or osmotic stress tolerance mechanisms [44,45]. Our results are in agreement with those of Beyaz [19], who observed an increase in root dry weight under water stress in vetch and a decrease in shoot weight in the PEG medium compared with the control medium. However, our work showed an average decrease in shoot dry weight of 47.9%, compared with Beyaz’s 11.5%. This difference may be because Beyaz [19] tested only one genotype and used a treatment with 10% PEG, while we have tested 26 genotypes and the PEG concentration was 20%. It should be noted that the different genotypes behave differently (Table S2) and that the growing medium also affects the dry weight of roots and shoots, with statistically significant differences between genotypes and interaction with the growing medium used (Table 3 and Figure 4), which agrees with the observations made by Bukan et al. [20] in soybean.

Plants that invest a greater proportion of resources in root development enhance their capacity for water and nutrient uptake, whereas those allocating more resources to shoot and leaf growth improve their ability to intercept light and assimilate CO_2_. Moreover, several studies [46,47,48] have demonstrated that biomass partitioning between roots and shoots is environmentally influenced and under genetic control. In some cultivated species, such as *Phaseolus vulgaris* and *Vigna radiata*, it has been shown that devoting more energy to root system development than to shoot growth improves drought resistance compared with plants with a lower root/shoot ratio [49,50]; in the same sense, Cabeza et al. [21] identified a QTL and five candidate genes for root/shoot dry weight ratio in barley. In our work, the ratio between the dry weight of roots and shoots varied according to the *in vitro* culture medium in which the seedlings are grown (Table 2). Thus, the mean value of the trait R0/S0 for the set of genotypes analysed was 0.75, whereas for R20/S20, it was 1.67. Again, it is worth noting that there were differences in the behaviour of the different genotypes (Table S1), and the interaction between genotype and culture medium was highly significant (Table 3 and Figure 4). Therefore, the difference in biomass distribution between roots and shoots among vetch genotypes depends on the environment in which they grow (C0 or C20) and can be explained by the plants’ ability to adapt their phenotype in response to drought as an environmental challenge. In our study, genotype V4 exhibited the lowest root-to-shoot dry weight ratio in both the C0 and C20 media, whereas genotypes V6 and V1 exhibited the highest ratios in the C0 and C20 media, respectively.

### 3.2. Field Results

It is important to note that the most economically valuable product of vetch is its seeds, which are primarily used in the production of animal feed [27]. Consequently, breeding programmes aimed at developing higher-yielding varieties typically begin by characterising seed yield across different genotypes. In the present study, the seed yield of 26 vetch genotypes under two contrasting environmental conditions was evaluated: natural conditions and drought conditions. The weight of 100 seeds and the weight of seeds per plant are the parameters we used to evaluate vetch grain yield. On average, both traits were 19.3% and 26.8% lower in the drought condition compared with the natural condition. In addition, as in *in vitro* culture experiments, there is considerable intergenotypic variability (Table S1 and Table 2), with highly productive genotypes such as V8 and others with very low productivity such as V17, as well as genotypes that are either strongly or minimally affected by drought conditions, such as V20 and V17, respectively (Figure 4). Moreover, we found strong positive correlations between NWS and DWS (r = 0.94), as well as between these two traits and DWP (r = 0.77), but not with NWP. Similar results were reported by Cakmakci et al. [51], who conducted a study of agronomic traits in a vetch collection aimed at analysing the heritability of various traits and identifying correlations among them. We have also observed interactions between genotype and environment for the trait of seed weight per plant, but not for 100 seed weight (Table 3 and Figure 4). This suggests that certain genotypes are more susceptible to water stress than others, as Alicandri et al. [43] already observed in common bean.

Gupta et al. [52] reported that total root length per unit root dry weight is a reliable trait for predicting drought tolerance in chickpea, which is defined as plant performance under conditions of low soil moisture content. In our study, the correlation analysis between parameters related to seedling root and shoot dry weight and seed production reveals some associations of notable applied interest. Specifically, positive correlations were found between R0, S0, and the S0/S20 ratio with NWS, DWS, and DWP (Figure 5). Therefore, it is possible to establish a relationship between the dry weight of seedling roots and shoots with seed yield in adult plants, which is highly relevant when evaluating large germplasm collections, as it enables substantial savings in time and resources.

### 3.3. Proline Analysis

Proline concentration has been shown to rise in plants under abiotic stress conditions, including water stress; however, it not clear that this accumulation is correlated with drought tolerance [11,53]. We have found that, in the vetch genotypes analysed, the proline concentration in roots is lower than in shoots, both for seedlings grown in C0 and C20 medium, although in the latter medium, the proline concentrations of both organs are much higher than those observed in C0 (Table 2). In addition, there is high intergenotypic variability (Table 4 and Table S1), and the GxM interaction is significant (Table 3, Figure 4).

Muktadir et al. [54] proposed using biochemical marker-based selection in bean breeding programmes as a faster way to develop new varieties. In the same species, Arteaga et al. [13] found a clear association between proline concentration and growth inhibition under salt and water stress conditions. However, the beneficial role of increased proline concentration is still under debate, and several studies have reported neutral or even detrimental effects, suggesting that, in certain cases, proline may represent a symptom of stress rather than an adaptive response that helps to mitigate it [11,55]. Our results indicate that the correlations between proline concentrations in the roots and shoots of seedlings and traits related to root and shoot dry weight, as well as agronomically relevant traits such as 100 seed weight and seed weight per plant, were generally inconclusive. Only a moderate correlation was observed between the S0/S20 ratio and RP20 (r = 0.5), along with two probably spurious correlations between NWP with SP0 (r = 0.42) and NWP with RP20 (r = 0.48).

These results are probably because the effect of drought on proline concentration in different plant organs varies according to genotype, intensity and duration of stress, and growth stage of the plant [7,56]. Regardless, the results obtained in our study suggest that proline concentration is not an effective predictor of drought tolerance in vetch, which corroborates the previously contradictory findings of other researchers. For example, Herrera Flores et al. [36] and Kusvuran and Dasgan [57] for *Phaseolus* and Hirarapa et al. [15] for chickpea found that more drought-tolerant genotypes accumulated more proline, whereas Morosan et al. [58] observed that less tolerant genotypes accumulated more proline in *Phaseolus*. In view of the results obtained in the proline study, we decided not to include proline concentration in the subsequent analyses because there were no clearly significant correlations with the traits related to agricultural production (weight of 100 seeds and weight of seeds per plant).

### 3.4. Principal Component Analysis

Previous research has addressed drought tolerance in vetch [9,10,31,59]. However, to the best of our knowledge, no study has evaluated tolerance to water stress in juvenile plants of different genotypes using multiple approaches while also relating these responses to seed yield, which is the main agronomic interest in this species. We conducted a PCA from the traits analysed but excluded those related to proline concentration in the roots and shoots of vetch seedlings. This analysis aimed at classifying the vetch genotypes according to biomass production and tolerance of water stress (Figure 6). The PC1 enables the separation of groups according to their biomass production in *in vitro* experiments, as well as the seed weight of plants cultivated in the field under natural conditions and with higher water stress imposed by the rain shelter. In PC2, the groups are separated mainly according to their drought tolerance as measured by the traits R0/R20 and S0/S20 and by the root/shoot dry weight ratio. Based on the PCA analysis, we proposed classifying vetch genotypes into six groups (A–F), which exhibit clear differences in the analysed traits but a high degree of homogeneity within each group, with low coefficients of variation (Table 5). Thus, group B (genotype V8) presents high biomass production in both *in vitro* and field conditions but shows high sensitivity to *in vitro* imposed water stress, as indicated by high R0/R20 and S0/S20 values. At the other extreme, group E includes a landrace from Iran (V17), another from Turkey (V18), and two from Spain (V20 and V25), characterised by low biomass production and low sensitivity to water stress. Group A (genotype V16) has the lowest biomass production and water stress tolerance, while groups C and D show intermediate–low values for both biomass production and water stress. Group F is characterised by medium-to-high yield and tolerance to water stress and includes the three commercial varieties as well as two local Spanish varieties (V23 and V24), which could serve as promising starting material for the development of new commercial cultivars.

## 4. Materials and Methods

### 4.1. Plant Material

A collection of 26 vetches (*V. sativa* L.) originating from five countries including 3 commercial varieties, 21 landraces, and 2 wild populations were provided by the National Plant Genetic Resource Centre of Spain (CRF, INIA/CSIC) (Table 6). This material was accessed via the International Treaty PGRFA (Plant Genetic Resources for Food and Agriculture) Standard Material Transfer Agreement

### 4.2. In vitro Experiments

#### 4.2.1. Culture Medium

Two culture media, designated as C0 and C20, were used. Both media consisted of ½ × Hoagland’s solution [60] and 0.75% Gelrite (Duchefa Biochemie™ Haarlem, The Netherlands). C20 medium additionally contained 20% polyethylene glycol-6000 (PEG-6000, Duchefa Biochemie™ London, United Kingdom). Both media were autoclaved at 120 °C for 20 min, and after cooling to 60 °C, they were poured into 9 cm diameter Petri dishes. The selection of the 20% PEG concentration was based on our previous (unpublished) studies on the vetch variety ‘Aitana,’ in which different PEG concentrations were tested, and the concentration chosen was the one that produced a 50% decrease in seedling root and shoot growth.

#### 4.2.2. Roots and Shoots Characterisation

Seeds of uniform size were selected from each genotype and surface-disinfected in a sodium hypochlorite solution (3:1 Domestos Unilever™, London, UK) for 15–20 min. Subsequently, the seeds were rinsed four times with sterile deionised water. Seeds of each genotype were distributed into six Petri dishes containing two filter papers moistened with 5 mL of sterile deionised water. The Petri dishes were then placed in a cold chamber at 4 °C for seven days. After this period, sixty seeds that started to germinate were transferred to six Petri dishes, three with C0 medium and three with C20 medium, and incubated in the dark at a constant temperature of 24 °C. Fifteen days after transplantation in Petri dishes, the developed plantlets were removed, and the roots and shoots were carefully separated from the culture medium. The roots and shoots of the 30 seedlings grown in C0 medium were placed in six glass tubes containing 10 roots or 10 shoots each. The same was performed with the roots and shoots of the 30 seedlings grown in C20 medium. The shoot and root samples were then dried in an oven at 70 °C for 16 h, after which the root dry weight and the shoot dry weight was obtained.

#### 4.2.3. Proline Determination

Following the previous protocol for seedling growth, the concentration of proline was quantified in three fresh roots and shoots of each vetch genotype grown *in vitro* in media with or without PEG, according to the method described by Bates et al. [61]. For proline extraction, 0.1 g of root or shoot tissue was homogenised in 1 mL of 3% (*w*/*v*) sulfosalicylic acid, followed by centrifugation at 13,000 rpm for 10 min. The supernatant was collected and mixed with acetic acid and acid ninhydrin in equal proportions, incubated at 97 °C for 30 min, and cooled on ice for 10 min, and its absorbance was measured at 520 nm. Proline concentration was quantified using a standard calibration curve and expressed in µg/mL. The root and shoot proline content was reported as µg/100 mg fresh weight tissue.

### 4.3. Vetch Seed Production in Field Experiments

Field studies were conducted at the ‘La Canaleja’ farm (Alcalá de Henares, Madrid (602 masl; 40°30′54″ N/03°18′42″ W)). Seeds were germinated in trays with peat on 10 October 2022 and grown in the greenhouse until 17 November 2022, when they were transplanted to the field. Plants of the 26 vetch genotypes were grown in an elemental plot of 6 plants per plot and three replicates in two environments: in the field under natural rain-feed conditions supplemented with 3 irrigation cycles of 6 l/m^2^ (N); and in a drought condition using a rain shelter since the beginning of flowering (D). After harvest, the weight of 100 seeds and the weight of seeds per plant were measured.

The data obtained from the *in vitro* and field experiments were used to annotate or calculate the traits shown in Table 1.

### 4.4. Statistical Analysis

The *in vitro* experiment was conducted as a completely randomised block design, with three replicates for both the root and shoot dry weight study (ten seedlings/replicate) and for the proline concentration study (one seedlings/replicate) and two treatments (control: medium C0; and water stress: medium C20). The field experiments were also conducted in a randomised block design with three replications of six plants each and two environments: natural field conditions (N) and drought conditions (D).

Genotype was treated as a fixed effect, while replicated, in *in vitro* growth medium, and field growing conditions were considered random effects. Prior to analysis of variance (ANOVA), data were transformed using a base-10 logarithm to meet the assumptions of normality. Post-hoc multiple comparisons were performed using Tukey’s honestly significant difference (HSD) test or LSD (least significant difference) test at a significance level of α = 0.05. Pearson’s correlation coefficients were calculated for the traits from the *in vitro* and field experiments. Statistical significance was determined at α = 0.05. A principal component analysis (PCA) was performed using the mean values of the traits. Broad-sense heritability in percentages (*h*^2^_%_) was estimated using the formula: *h*^2^_%_ = σ^2^G/(σ^2^G + (σ^2^ ε/r))*100, where *σ^2^ G* and *σ^2^ ε* are the genotypic and error variance and *r* denotes the number of replicates. Statistical analyses were performed using the ‘Statgraphics Centurion 19’ (Statpoint Technologies, Warrenton, VA, USA) software.

## 5. Conclusions

This study revealed substantial variability among vetch accessions for all evaluated traits, with distinct genetic responses to drought stress observed both *in vitro* and under field conditions. Broad-sense heritability exceeded 60% for most traits, indicating strong genetic control and promising prospects for selection. Root and shoot dry weights of *in vitro* seedlings, evaluated under both control and water stress conditions, were identified as reliable predictors of seed yield in field-grown plants, offering an efficient screening tool for drought tolerance. Conversely, proline accumulation, although induced under stress, showed no clear correlation with agronomically relevant traits and was not a suitable marker. Genotypes with superior biomass production and drought tolerance emerged as valuable candidates for breeding programmes, while hybridisation between contrasting genotypes could facilitate the detection of genomic regions associated with drought tolerance. These results provide a framework for breeding strategies aimed at improving drought adaptation in vetch, contributing to resilient and sustainable cropping systems.

## Figures and Tables

**Table 1:** List of the traits used in the study together with a description of each of them.

Trait	Description
R0	Root dry weight of seedlings grown in C0 medium (mg)
S0	Shoot dry weight of seedlings grown in C0 medium (mg)
R20	Root dry weight of seedlings grown in C20 medium (mg)
S20	Shoot dry weight of seedlings grown in C20 medium (mg)
RP0	Proline concentration in roots of seedlings grown in C0 medium (µg/100 mg tissue)
SP0	Proline concentration in shoots of seedlings grown in C0 medium (µg/100 mg tissue)
RP20	Proline concentration in roots of seedlings grown in C20 medium (µg/100 mg tissue)
SP20	Proline concentration in shoots of seedlings grown in C20 medium (µg/100 mg tissue)
NWS	Weight of 100 seeds of plants grown under rainfed conditions (gr)
DWS	Weight of 100 seeds of plants grown under drought conditions (gr)
NWP	Seed weight per plant grown under rainfed conditions (gr)
DWP	Seed weight per plant grown under drought conditions (gr)
R0/S0	Relationship between root dry weight and shoot dry weight of seedlings grown in C0 medium
R20/S20	Relationship between root dry weight and shoot dry weight of seedlings grown in C20 medium
R0/R20	Ratio between the dry weight of roots of seedlings grown in C0 medium and in C20 medium
S0/S20	Ratio between the dry weight of shoots of seedlings grown in C0 medium and in C20 medium
RP0/SP0	Ratio between root proline concentration and shoot proline concentration in seedlings grown in C0 medium
RP20/SP20	Ratio between root proline concentration and shoot proline concentration in seedlings grown in C20 medium
RP0/RP20	Ratio between the proline concentration of roots of seedlings grown in C0 medium and in C20 medium
SP0/SP20	Ratio between the proline concentration of shoots of seedlings grown in C0 medium and in C20 medium
NWS/DWS	Ratio between the weight of 100 seeds of plants grown under rainfed and drought conditions, respectively
NWP/DWP	Ratio between the seed weight per plants grown under rainfed and drought conditions, respectively

**Table 2:** Mean, minimum and maximum values, standard deviation (SD), coefficient of variation (CV), and broad heritability in percentage (h^2^%) for each of the traits analysed in the *in vitro* culture experiments and in the field trials, considering the 26 vetch genotypes. The meaning of the abbreviations and the description of each trait is shown in Table 1.

	Traits																					
	R0 (mg)	S0 (mg)	R20 (mg)	S20 (mg)	RP0 (µg/100 mg Tissue)	SP0 (µg/100 mg Tissue)	RP20 (µg/100 mg Tissue)	SP20 (µg/100 mg Tissue)	NWS (gr)	DWS (gr)	NWP (gr)	DWP (gr)	R0/ S0	R20/ S20	RP0/ SP0	RP20/ SP20	R0/ R20	S0/ S20	RP0/ RP20	SP0/ SP20	NWS/ DWS	NWP/ DWP
Mean	5.99	7.99	6.97	4.16	15.96	27.88	34.80	64.44	6.32	5.10	14.26	10.44	0.75	1.67	0.58	0.56	0.86	1.92	0.55	0.49	1.25	1.45
Min	3.39	4.62	2.46	2.74	9.07	16.49	5.58	18.36	3.81	2.67	3.98	4.82	0.58	0.84	0.40	0.17	0.52	1.39	0.26	0.24	1.06	0.67
Max	11.90	15.37	16.32	7.28	24.32	42.52	62.53	107.3	10.51	9.47	21.64	19.56	0.97	2.55	0.91	1.21	1.63	2.78	1.68	1.15	1.43	2.91
SD	1.96	2.40	3.23	1.01	4.60	6.97	13.41	24.70	1.51	1.33	3.98	3.30	0.11	0.51	0.12	0.20	0.28	0.36	0.32	0.21	0.11	0.52
CV	32.79	30.10	46.31	24.29	28.85	25.01	38.53	38.34	23.39	26.04	26.04	31.57	14.90	31.09	20.15	35.61	29.75	18.40	58.27	43.84	8.61	35.62
h^2^ (%)	90.3	87.7	87.7	87.8	65.9	78.6	73.1	77.4	82.8	63.8	63.9	68.2	53.7	74.5	30.70	41.50	70.6	61.9	78.6	63.6	50.4	41.5

**Table 3:** Results of the multifactorial ANOVAs for genotype and *in vitro* medium growth or field growth based on the following dependent traits: root dry weight (R), shoot dry weight (S), root-to-shoot dry weight ratio (R/S), proline concentration in roots (RP) and in shoots (SP), the weight of 100 seeds (WS), and the average seed weight per plant (WP). The medium (M) corresponds to C0 and C20 medium for *in vitro* experiments and rainfed (N) or drought (D) conditions for field experiments. Sum of squares (SS), degrees of freedom (d.f.). G × M represents the interaction between genotype and *in vitro* medium or field condition. *p*-values: -: not significant; ***: *p* < 0.001.

Trait	Source of Variation	d.f.	Sum Square	Mean Square	F-Ratio
R	Genotype (G)	25	3.4161	0.1366	41.99 ***
	Medium (M)	1	0.0675	0.0675	20.75 ***
	GxM	25	0.6216	0.0248	7.64 ***
	Residual	104	0.3384	0.0032	
	Total	155	4.4436		
S	Genotype (G)	25	1.5246	0.0610	30.94 ***
	Medium (M)	1	3.0190	3.0190	1531.6 ***
	GxM	25	0.2254	0.0090	4.57 ***
	Residual	104	0.2050	0.0019	
	Total	155	4.9741		
R/S	Genotype (G)	25	1.2561	0.0502	12.60 ***
	Medium (M)	1	3.9894	3.9894	1000.30 ***
	GxM	25	0.4785	0.0191	4.80 ***
	Residual	104	0.4148	0.0039	
	Total	155	6.1387		
RP	Genotype (G)	25	3.9027	0.1561	23.18 ***
	Medium (M)	1	3.7016	3.7016	549.75 ***
	GxM	25	1.4189	0.0567	8.43 ***
	Residual	104	0.7002	0.0067	
	Total	155	9.7235		
SP	Genotype (G)	25	2.7394	0.1095	19.88 ***
	Medium (M)	1	4.3797	4.3797	794.56 ***
	GxM	25	1.2693	0.0507	9.21 ***
	Residual	104	0.5732	0.0055	
	Total	155	8.9618		
RP/SP	Genotype (G)	25	1.1339	0.0436	3.78 ***
	Medium (M)	1	0.0425	0.0425	3.54 -
	GxM	25	1.2801	0.0512	4.27 ***
	Residual	104	3.7031	0.0120	
	Total	155			
WS	Genotype (G)	25	1.6358	0.0654	14.88 ***
	Field growth (M)	1	0.3914	0.3914	89.03 ***
	GxM	25	0.0624	0.0024	0.57 -
	Residual	104	0.4572	0.0044	
	Total	155	2.5468		
WP	Genotype (G)	25	2.3932	0.0957	12.69 ***
	Field growth (M)	1	0.7181	0.7181	95.17 ***
	GxM	25	0.8242	0.0329	4.37 ***
	Residual	104	0.7847	0.0075	
	Total	155	4.7202		

**Table 4:** Weights for each trait in the two first components in the PCA analysis performed on vetch genotypes. The meaning of the abbreviations and the description of each trait is shown in Table 1.

Trait	PC1	PC2
R0	0.3956	−0.0320
S0	0.3804	0.0880
R20	0.3300	−0.3467
S20	0.3125	−0.1813
NWS	0.3524	0.2346
DWS	0.3578	0.1961
NWP	0.0254	0.1025
DWP	0.3070	0.2593
R0/S0	0.0755	−0.2930
R20/S20	0.2262	−0.3957
R0/R20	−0.1051	0.4938
S0/S20	0.1675	0.3917
NWS/DWS	−0.0825	0.1198
NWP/DWP	−0.2051	−0.1123

**Table 5:** Statistical summary of the traits analysed in the 7 groups of genotypes established after PCA analysis. In the rows of homogeneous groups for each trait, different letters represent a significant difference at *p* < 0.05. The meaning of the abbreviations and the description of each trait is shown in Table 1.

Group Name		Trait													
		R0	S0	R20	S20	NWS	DWS	NWP	DWP	R0/ S0	R20/S20	R0/ R20	S0/ S20	NWS/ DWS	NWP/ DWP
A	Mean	4.00	6.43	2.46	2.74	5.46	4.27	12.78	8.97	0.63	0.90	1.63	2.34	0.71	1.45
	SD	0.35	1.04	0.20	0.13	0.36	0.60	1.20	1.18	0.11	0.03	0.04	0.34	0.12	0.32
	CV	0.09	5.61	0.08	0.05	0.07	0.14	0.09	0.13	0.18	0.04	0.02	0.14	0.17	0.22
	Min	3.61	7.60	2.23	2.60	5.12	3.75	12.07	7.80	0.54	0.86	1.59	2.00	0.59	1.19
	Max	4.27	2.00	2.59	2.84	5.83	4.92	14.17	10.15	0.76	0.91	1.67	2.68	0.83	1.82
Homogeneous group		a	ab	a	a	a	abc	ab	abc	a	a	a	cd	d	b
B	Mean	11.90	15.37	10.03	5.53	10.51	9.47	13.04	19.56	0.78	1.82	1.20	2.78	0.44	0.67
	SD	0.83	0.88	1.18	0.10	1.50	1.06	1.08	1.29	0.07	0.25	0.22	0.18	0.10	0.09
	CV	0.07	14.71	0.12	0.02	0.14	0.11	0.08	0.07	0.09	0.14	0.18	0.06	0.22	0.14
	Min	10.97	16.36	8.88	5.43	8.78	8.44	11.80	18.10	0.73	1.58	0.98	2.61	0.35	0.57
	Max	12.58	1.66	11.25	5.64	11.41	10.55	13.76	20.57	0.86	2.07	1.42	2.96	0.54	0.76
Homogeneous group		e	d	de	c	d	e	ab	d	ab	cd	bc	d	abc	a
C	Mean	4.82	7.19	4.58	3.68	6.08	4.71	14.36	9.57	0.69	1.25	1.09	1.97	0.57	1.59
	SD	0.82	1.52	0.99	0.57	0.75	0.88	3.99	2.58	0.15	0.24	0.27	0.39	0.14	0.59
	CV	0.17	4.73	0.22	0.16	0.12	0.19	0.28	0.27	0.21	0.19	0.24	0.20	0.25	0.37
	Min	2.85	11.04	2.93	2.71	4.69	3.17	6.30	4.87	0.40	0.78	0.69	1.24	0.31	0.68
	Max	6.03	6.30	6.62	4.82	7.41	6.79	23.27	16.97	1.02	1.71	1.70	2.93	0.82	3.13
Homogeneous group		ab	b	b	b	b	b	b	b	a	b	b	bc	cd	b
D	Mean	6.23	7.89	6.29	3.99	6.79	5.59	16.71	12.60	0.79	1.64	1.00	2.03	0.51	1.42
	SD	1.20	1.26	1.03	0.75	0.60	1.28	4.44	2.89	0.12	0.45	0.18	0.38	0.12	0.61
	CV	0.19	5.94	0.16	0.19	0.09	0.23	0.27	0.23	0.15	0.27	0.18	0.19	0.24	0.43
	Min	4.43	10.44	4.00	2.77	5.75	3.68	8.10	8.30	0.62	0.97	0.78	1.35	0.31	0.64
	Max	8.93	4.50	8.48	5.16	8.10	8.19	24.50	18.33	0.99	2.56	1.47	2.62	0.71	2.95
Homogeneous group		c	b	c	b	c	cd	b	c	b	c	b	bc	bc	b
E	Mean	5.31	6.24	7.96	4.14	4.18	3.57	11.82	7.24	0.86	1.98	0.69	1.53	0.46	1.71
	SD	0.89	1.16	1.81	0.83	0.59	0.85	5.79	2.57	0.09	0.50	0.19	0.24	0.14	0.90
	CV	0.17	4.41	0.23	0.20	0.14	0.24	0.49	0.36	0.10	0.25	0.27	0.16	0.30	0.53
	Min	3.92	7.73	5.63	3.10	3.35	1.92	3.37	3.77	0.72	1.05	0.49	1.23	0.31	0.62
	Max	6.56	3.32	10.74	5.37	5.03	4.37	20.71	12.60	1.02	2.54	1.11	2.09	0.77	3.53
Homogeneous group		b	a	d	b	a	a	a	a	b	d	a	a	b	b
F	Mean	7.83	9.92	11.92	5.32	7.55	5.91	14.11	11.07	0.79	2.27	0.67	1.86	0.36	1.31
	SD	1.80	2.43	2.98	1.19	1.44	1.01	4.60	3.55	0.07	0.42	0.15	0.11	0.08	0.34
	CV	0.23	6.41	0.25	0.22	0.19	0.17	0.33	0.32	0.09	0.19	0.22	0.06	0.22	0.26
	Min	4.81	14.73	7.90	3.65	5.08	3.96	6.39	5.08	0.68	1.62	0.48	1.68	0.25	0.66
	Max	10.94	8.33	17.21	7.42	10.10	7.60	21.85	17.82	0.95	2.97	0.93	2.08	0.51	1.96
Homogeneous group		d	c	e	c	c	d	ab	bc	b	d	a	b	a	b

**Table 6:** Vetch genotypes analysed with an indication of the working code, Spanish Genebank number or variety name, geographical origin, and type of material.

Working Code	Variety Name or Spanish Genebank Number	Country	Local Origin	Type of Plant Material
V1	AITANA	SPA		commercial variety
V2	BGE000529	GRC	Vromovrisi (Peloponissos)	landrace
V3	BGE000587	IRN	Isfahan_Arak	landrace
V4	BGE000600	IRN	Firuz Kuh (Teheran)	landrace
V5	BGE001163	SPA	Guareña (Badajoz)	landrace
V6	BGE004356	SPA	Tolox (Málaga)	landrace
V7	BGE004375	SPA	Mala (Granada)	landrace
V8	BGE005449	SPA	Andujar (Jaen)	landrace
V9	BGE007269	SPA	Socuellamos (Ciudad Real)	landrace
V10	BGE014945	SPA	Valdeganga (Albacete)	landrace
V11	BGE014946	SPA	Iniesta (Cuenca)	landrace
V12	BGE022207	SPA	Mao-Mahon (Islas Baleares)	landrace
V13	BGE022757	ITA	Caltavuturo (Palermo)	landrace
V14	BGE025608	SPA	Valdelacasa de Tajo (Cáceres)	landrace
V15	BGE022210	SPA	Benilloba (Alicante)	landrace
V16	BGE026275	SPA	Cazorla (Jaen)	landrace
V17	BGE000418	IRN	Borujerd_Korramabad (Lorestan)	landrace
V18	BGE000528	TUR	Kurtkoy (Istambul)	landrace
V19	BGE014901	SPA	Guadarrama (Madrid)	wild population
V20	BGE016970	SPA	Madrid	wild population
V21	BGE029065	SPA	Sevilla	landrace
V22	SENDA	SPA		commercial variety
V23	BGE004289	SPA	Pinos Puente (Granada)	landrace
V24	BGE004419	SPA	Cadiar (Granada)	landrace
V25	BGE027063	SPA	Torvizcon (Granada)	landrace
V26	VERDOR	SPA		commercial variety

## Data Availability

Dataset available on request from the authors.

## Supplementary Materials

The following supporting information can be downloaded at: https://www.mdpi.com/article/10.3390/plants14213376/s1, Table S1: Mean and standard deviation (SD) of each of the traits analysed in each of the vetch genotypes studied; Table S2: Results of Tukey’s HSD tests between vetch genotypes for each of the 22 traits studied. For each of the traits, genotypes with the same letter do not show statistically significant differences (*p* < 0.05).
