# Supplementary material for: Combining in vitro and Field Studies to Predict Drought Tolerance in Vicia sativa L. Genotypes
Source: Plants (Basel). 2025 Nov 4;14(21):3376. doi: 10.3390/plants14213376 (PMC12609475; doi:10.3390/plants14213376)
Supplement: Supplementary file 1 [file plants-14-03376-s001.zip › Table S2.pdf]

Table S2.- Results of Tukey's HSD tests between vetch genotypes, for each of the 22 traits studied. For each of the trait, genotypes with the same letter do not show statistically significant differences ($p< 0.05$).

Genotype Code	Trait																					
	R0	S0	R20	S20	RP0	SP0	RP20	SP20	NWS	DW S	NWP	DW P	R0/ S0	R20/ S20	RP0/ SP0	RP20/ SP20	R0/ R20	S0/ S20	RP0/ RP20	SP0/ SP20	NWS/ DWS	NWP/ DWP
**V1**	bcde	bcdef	fg	cdefg	cdef	abcd	bcdefg	efgh	def	bcde	bcdef	bcde	abcd	k	ab	bcd	a	abcd	bcde	abce	a	abc
**V2**	bcd	bcde	abcd	abcdef	cdef	bcdefgh	cdefgh	gh	def	abcd	bcdef	bcd	abcd	abcd	ab	abcd	fgh	abcde	abcd	ab	cde	abcd
**V3**	abc	ab	cde	abcdef	def	ghi	cdefgh	efgh	bcde	abcd	def	abcd	abcd	cdefghijk	ab	bcd	abcdef	abcd	cde	bcde	abcde	bcd
**V4**	abcd	bcdefg	bcde	fgh	def	efghi	cdefgh	bcdefgh	def	bcd	bcd	bcd	a	abc	ab	bcde	defgh	abcd	abcd	cde	cde	abc
**V5**	bcde	cdefg	bcde	bcdefg	abc	abcde	defgh	efgh	efg	abcd	bcdef	cde	ab	abcdefg	ab	bcde	cdefgh	cde	ab	abcd	abcde	abc
**V6**	bcdef	ab	abc	abc	bcdef	fghi	bcdefgh	gh	bcde	abc	def	bcd	d	abcdef	ab	abc	gh	abcde	abcd	abcd	e	bcd
**V7**	abc	ab	bcde	abcd	abcde	defghi	defgh	efgh	cdef	abcd	def	bcd	abcd	abcdefgh	a	bcde	bcdefgh	abcd	abcd	abcd	bcde	cd
**V8**	h	i	fg	hi	bcdef	defghi	bcdefgh	cdefgh	h	e	bcdef	e	abcd	defghijk	ab	bcde	efgh	e	abcd	abcde	abcde	a
**V9**	bcd	bcdefg	bcd	ab	abcdef	bcdef	efgh	defgh	def	bcde	cdef	bcd	abcd	abcdefg	ab	cde	fgh	de	abcd	abcd	abcde	abcd
**V10**	defg	bcdef	def	defg	cdef	cdefghi	bcdef	bcdefgh	def	bcde	bcde	bcde	abcd	cdefghijk	ab	bcd	bcdefgh	abcd	cde	abcd	bcde	abc
**V11**	bcdefg	bcdefg	ef	abcd	abcd	bcdef	efgh	bcdefg	efg	bcde	f	bcd	abcd	ghijk	ab	de	abcdef	cde	ab	abcd	abc	bcd
**V12**	efg	bcdefg	ef	abcdef	ab	abcd	cdefgh	efgh	def	bcd	bcdef	de	bcd	fghijk	Ab	bcde	defgh	cde	a	abcd	abcde	abc
**V13**	bcd	bcdef	bcde	fgh	abcdef	bcdef	bcde	bcde	efgh	cde	ef	cde	abcd	abcde	ab	bcde	bcdefg	abc	abcd	abcde	bcde	abcd
**V14**	a	ab	ab	defg	abcd	cdefghi	b	a	def	abcd	cdef	cde	a	a	Ab	bcde	bcdefgh	ab	def	de	de	abcd
**V15**	bcd	bcdefg	bcde	defg	abc	cdefghi	bcdefg	bcde	def	bcde	bcdef	ab	abcd	abcdef	a	bcde	bcdefgh	abcde	abcd	e	abcde	cd
**V16**	ab	abcd	a	a	abcd	abcd	efgh	bcd	bcde	abcd	bcdef	bcd	abc	ab	ab	e	h	cde	abc	de	de	abcd
**V17**	abc	a	ef	abcde	a	a	a	ab	ab	ab	a	a	cd	fghijk	ab	a	abcd	a	e	abcd	abcde	ab
**V18**	defg	bcde	fg	gh	bcdef	cdefghi	bcdefg	bcdefgh	abc	abcd	bcde	bcde	cd	efghijk	ab	bcde	abcde	a	abcd	bcde	abcde	abc
**V19**	efg	fgh	ef	gh	ef	bcdefg	fgh	fgh	def	bcd	cdef	cde	abcd	abcdef	b	bcde	defgh	abcde	abcd	abc	bcde	abcd
**V20**	bcdef	bcdef	ef	efgh	abc	abc	a	a	a	a	def	ab	abcd	cdefghij	ab	bcd	abcdef	abc	de	e	abcde	d
**V21**	bcdef	efg	ef	fgh	abcdef	abcd	bcdef	bcdef	abcd	abc	bc	abc	a	bcdefghi	ab	bcde	abcdefg	bcd	abcd	abcd	abcd	abc
**V22**	fgh	gh	fg	hi	a	ab	bc	efgh	def	abcd	ab	ab	abcd	defghijk	ab	ab	abcdef	abcde	abcd	a	abcde	abcd
**V23**	defg	defg	gh	gh	abcd	abc	bcd	abc	gh	de	cdef	cde	abcd	ijk	ab	bcde	abcd	abcde	cde	cde	abc	abc
**V24**	efg	efg	gh	gh	f	i	h	fgh	def	bcde	def	cde	abcd	jk	ab	bcde	ab	abcde	abcd	abcd	abc	abcd
**V25**	bcd	abc	fg	bcdefg	def	hi	cdefgh	defgh	abc	abc	cdef	abc	abcd	jk	ab	bcde	a	abcd	cde	de	Ab	bcd
**V26**	gh	hi	h	i	ef	defghi	gh	h	fgh	cde	def	cde	abcd	hijk	ab	bcde	abc	abcde	abcd	abcd	abc	abcd
